# Voices from the frontline: counsellors' perspectives on TB/HIV collaborative activities in the Northwest Region, Cameroon

**DOI:** 10.1186/1472-6963-11-328

**Published:** 2011-11-30

**Authors:** Barnabas N Njozing, Kerstin E Edin, Miguel San Sebastián, Anna-Karin Hurtig

**Affiliations:** 1 St. Mary Soledad Catholic Hospital, Mankon, Bamenda, P.O.Box 157, Cameroon; 2 Department of Public Health and Clinical Medicine, Unit of Epidemiology and Global Health, Umeå University, Umeå, 901 85, Sweden; 3 Swedish Research School for Global Health, Umeå University, 901 85, Umeå, Sweden; 4 Umeå Centre for Gender Studies, Umeå University, Sweden

## Abstract

**Background:**

The overlapping epidemiology of tuberculosis (TB) and human immunodeficiency virus (HIV) infections prompted the World Health Organisation in 2004 to recommend collaboration between national TB and HIV programmes. The goal of this collaboration is to decrease the burden of both infections in the population. This policy was subsequently adopted by the national TB and HIV programmes in Cameroon with TB and HIV nurses/counsellors acting as frontline implementers of the collaborative activities in the 10 regions of the country.

**Methods:**

Qualitative research interviews were conducted with 30 nurses/counsellors in four approved treatment centres providing comprehensive TB and HIV/AIDS services in the Northwest region of Cameroon. The aim was to explore their experiences in counselling, in delivering joint TB and HIV services, and the constraints to effective collaboration between TB and HIV services. To complement the findings from the counsellors' interviews, as part of an emergent design, further interviews with 2 traditional healers and non-participant observations in two HIV support group meetings were conducted.

**Results:**

According to the respondents, counselling was regarded as a call to serve humanity irrespective of the reasons for choosing the profession. In addition, the counselling training and supervision received, and the skills acquired, have altogether contributed to build patients' trust in the healthcare system. Teamwork among healthcare workers and other key stakeholders in the community involved in TB/HIV prevention and control was used as a strategy to improve joint service delivery and patients' uptake of services. Several constraints to effective collaboration between TB and HIV services were identified, including shortage of human resources, infrastructure and drug supplies, poor patients' adherence to treatment and the influence of traditional healers who relentlessly dissuade patients from seeking mainstream medical care.

**Conclusions:**

In order to achieve a sustainable collaboration between TB and HIV services, adequate planning, investment and strengthening of the health system including human resources, infrastructure and ensuring uninterrupted supplies of medicines are essential. A multidisciplinary approach to service delivery particularly focusing on harnessing the enormous potentials of traditional healers in TB/HIV prevention and control would also be indispensible.

## Background

The human immunodeficiency virus (HIV) epidemic has contributed to the upsurge of tuberculosis (TB) infections with TB being the most common opportunistic infection in people living with HIV/AIDS (PLWHA), and the leading cause of death amongst PLWHA in Africa [1]. Because of the overlapping epidemiology of both infections, the World Health Organisation (WHO) recommended collaboration between national TB and HIV programmes in 2004 [2]. The goal of this collaboration is to reduce the burden of TB and HIV in populations affected by both infections, and has as its objectives: i) establishing mechanisms for collaboration between national TB and HIV programmes, ii) decreasing the burden of TB in PLWHA, and iii) decreasing the burden of HIV in people with TB [2].

Concerning establishing mechanisms for collaboration, WHO suggests that national TB and HIV programmes create a joint coordinating body that is responsible for planning, implementing, supervising and monitoring collaborative activities from the central down to the operational level. The joint coordinating body may either work together to produce joint TB/HIV plans or they may introduce TB/HIV components into each of the two national TB and HIV control plans [3]. In connection with decreasing the burden of HIV in people with TB, WHO since 2007 recommended that counselling and testing for HIV should be offered routinely by healthcare providers to all TB patients in areas of generalised HIV epidemic, i.e. where HIV prevalence is consistently over 1% in pregnant women [4]. This strategy will facilitate patients' access to HIV services. Similarly, as regards decreasing the burden of TB in PLWHA, WHO has recommended intensified TB-case finding and treatment in HIV programmes, in addition to providing Isoniazid to prevent the development of active TB in HIV patients found to have latent TB infection [2].

Since the 90s, the fight against TB and HIV/AIDS has been one of the national priorities in Cameroon and both programmes are organised into three levels: central, regional and peripheral [5]. The national AIDS control committee is the official body responsible for coordinating and managing the national AIDS control programme throughout the country. The central technical committee acts as its executive organ. At the intermediate level is the regional technical committee situated in all the 10 regions of the country, which together with the local committees at the peripheral level is responsible for implementing the activities defined at the central level. The Cameroon TB control programme, recognised as a national priority in 2002, defines the general objectives of the programme at the central level and the regional level organises, coordinates, monitors and evaluates TB control in the region. The peripheral level includes the health districts that constitute the primary structure of the TB programme which are responsible for TB case finding, treatment, and keeping the TB registers [5].

The country began establishing mechanisms for TB and HIV collaborative activities in 2004. A TB/HIV working group exists at the central level, consisting of members from both national TB and HIV programmes. The body is responsible for developing policies regarding collaborative activities. The regional TB and HIV units under the supervision of the regional delegations of public health are charged with implementing, monitoring and supervising these activities at the level of the various treatment centres in the regions.

TB and HIV nurses/counsellors are at the frontline in implementing TB and HIV collaborative activities as defined by the TB/HIV working group at the treatment centres throughout the national territory, and to a large extent determine how co-infected patients access and adhere to TB/HIV services. Since collaboration between TB/HIV programmes is a relatively new initiative with a dearth of knowledge, this study was conducted among TB and HIV nurses/counsellors offering comprehensive TB and HIV/AIDS services in four hospitals in the Northwest region of Cameroon. The purpose was to explore their experiences in counselling, in delivering joint TB and HIV/AIDS services, and ascertaining the constraints to effective collaboration between TB and HIV/AIDS services. Findings from this study are intended to improve the knowledge base, enhance TB and HIV/AIDS collaborative activities and patients' access to services which will eventually maximise the health and wellbeing of the general population.

## Methods

### Study setting

Located in Central Africa, Cameroon has an estimated population of 18 million inhabitants and a landmass of 475 440 km^2^. The country is divided into 10 regions with English and French being the official languages. This study was carried out in the Northwest region with Bamenda being its capital. The region comprises 7 administrative divisions with a population of over 2 million that is predominantly English speaking. The region has the highest HIV prevalence (8.7%) in the country (national prevalence is 5.5%). TB and HIV co-infection therefore poses an enormous threat to the population in the region and the country in general. There are presently 13 HIV/AIDS approved treatment centres [6] and 21 TB diagnostic and treatment centres in the region. Four hospitals which have both approved TB and HIV/AIDS treatment centres affiliated to them, and offer comprehensive TB/HIV services were purposefully selected for this study as a follow-up to previous studies evaluating TB/HIV collaborative activities in the region [7,8]. The selection of these centres was based on their accessibility, the diversity of patients they receive since they all serve both rural and urban populations, the similarity in patient load, and in the services provided. Moreover, they are the best centres in the region in terms of human, financial resources and infrastructure compared to the other treatment centres. These centres also act as referral centres in the region in terms of HIV management since they are equipped with functional CD4 count machines (BD FACSCount flow cytometer or Guava EasyCD4) required to monitor HIV patients' immune status. One of the centres also provides second-line antiretroviral treatment (ART) in addition to first-line regimens in the entire region. Three of the centres are faith-based hospitals and one is a public hospital.

### TB/HIV care

Screening, diagnosis, treatment and follow-up of all TB and HIV/AIDS patients are authorised only in approved TB and HIV treatment centres in the country, and are based on standardised guidelines issued by the national TB and HIV control programmes. However, the model of care for both infections is dependent on the individual centre's infrastructure and human/material resources available. In most faith-based centres, both units are co-located, and staff from both units function in parallel in delivering joint services. In addition, it is usually the same physician who oversees the management of both TB and HIV patients. Meanwhile in public centres, TB and HIV units are located apart but facilities for cross-referral exist. Furthermore, a therapeutic committee exists comprising of staff from both units that meet at least weekly to review co-infected patients' treatment regimens and progress.

At the TB centres, screening for TB is based on clinical signs and symptoms, and final diagnosis is by sputum microscopy although facilities for sputum cultures exist in some treatment centres. TB treatment, which is free of charge for all patients, normally lasts 6 months; 2 months intensive phase with Isoniazid, Rifampicin, Ethambutol and Pyrazinamide, followed by 4 months continuous phase with Isoniazid and Rifampicin. At the HIV units, screening for HIV is based on the clinical presentation and HIV rapid diagnostic test after the patients have been duly counselled and voluntary informed consent obtained. All patients suspected of having TB from the HIV units are referred to the TB unit for diagnosis, treatment and follow-up. Likewise, all newly diagnosed TB patients with an unknown HIV status are routinely offered HIV counselling and testing which is free of charge. This could either be performed by the TB nurses/counsellors in the TB unit or the patients are referred to the HIV unit depending on the treatment centre's routine and workload. All TB patients found to be co-infected with HIV are referred to the HIV unit for follow-up biological and immunological tests. All TB patients co-infected with HIV should be placed on Co-trimoxazole preventive (CPT) and ART which have been provided free of charge since May 2007. ART treatment is based on the national and WHO treatment guidelines [9]. The decision to initiate ART is either taken at the level of the attending physician or by a therapeutic committee (depending on the treatment centre in question). At the time of data collection for this study, modalities were still being drafted to scale up Isoniazid preventive therapy, intensification of TB diagnosis among PLWHA and in cluster populations like schools and prisons nationwide.

### Study design, sampling and data collection

Qualitative research interview was chosen as the most appropriate method of data collection since we focused on the participants' perceptions and experiences in counselling, in delivering joint TB and HIV services, and on the constraints to effective collaboration. Cognisant of the fact that TB and HIV/AIDS are very sensitive topics in the region, we aimed to explore how the participants understood and conducted themselves during service delivery, and when faced with constraints. Additionally, we used the interviews to further investigate findings from previous studies conducted in the region [7,8].

TB and HIV nurses/counsellors from all the study sites were purposively selected in order to capture their different perspectives regarding TB and HIV collaborative activities. They were approached and asked about their willingness to participate in the study and all expressly accepted to be interviewed. An interview guide with open-ended questions was developed based on findings from previous studies in the region [7,8], and also included items from WHO's recommendations for collaborative activities [2]. The first author performed the interviews which covered the following topics: the participants' background information, the reasons for becoming a counsellor, the nature of the training received, the counselling experience including the type of supervision/support available for counsellors, the benefits and constraints to effective collaboration between TB/HIV units, and suggestions for improvement of services. Based on preliminary comparative analysis of 30 conducted interviews, approximately 7 in each of the study sites, no new important topics emerged that in relation to the research question requested elaboration. It was therefore decided that further interviews with the nurses/counsellors would probably not yield much more additional knowledge [10].

A joint interview session was also conducted with two traditional healers; the president and the secretary general of the traditional healers' association operating within the same locality as one of the study hospitals. The rationale for selecting these healers being that they were influential in the collaboration that existed between members of their association and the study hospital in question pertaining to HIV prevention and referral of patients suspected of being infected with HIV to the treatment centre. All the interviews were conducted in either English or Pidgin English (a local language adapted from English) from September to December 2009. Each of the interviews lasted between 45-90 minutes and they were tape-recorded and transcribed verbatim by the first author. The tapes and transcripts were de-identified to safeguard the participants' identity.

Finally, with allowance to an emergent design in the data collection process, the first author assisted by two counsellors participated as an observer in two support group meetings for PLWHA. This was done to get a better understanding of the support services available to these patients, as mentioned in the interviews with some of the counsellors. Field notes were taken during and after these sessions.

### Data Analysis

**Figure 1:**
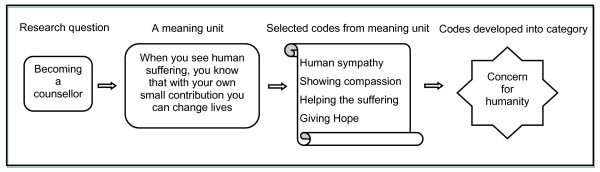
**An example of the analytical process**.

**Figure 2:**
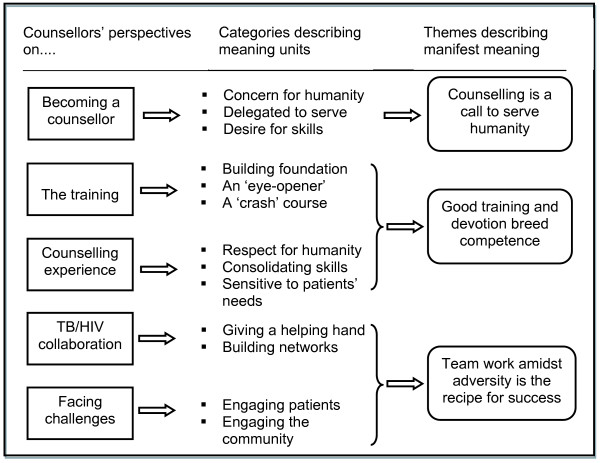
**Themes describing counsellors' perspective on TB/HIV collaboration**.

Qualitative Content Analysis [11] was used in the data analysis process. The focus was mainly on the manifest content of the texts which describes the nurses/counsellors' perspectives regarding counselling and collaboration between TB and HIV services. Initially, the interview texts were read through several times to get an in-depth understanding of the participants' perspectives regarding the interview topics. The texts were then sorted into five major content areas: i) the reasons for becoming counsellors, ii) the type of training received, iii) the counselling experience, iv) the nature of the collaboration between TB and HIV programmes and v) the constraints faced in the collaboration. Later, meaning units which represent a group of statements relating to the same central meaning were selected on which codes were manually assigned to them while maintaining the practice of constant comparison. The codes were then grouped together based on their similarities or differences into categories. An example of this process is illustrated in Figure 1. Finally, three themes were developed from the categories that described the manifest meaning (Figure 2). The observations and notes from the support group meetings and the interviews with the traditional healers were used to substantiate the findings from the interviews with the counsellors.

### Trustworthiness of the study

Several measures were employed before and during data collection/analysis to ensure trustworthiness of the study. Appropriate literature regarding the study objectives were reviewed combined with findings from earlier studies [7,8] in the region to ensure reliability and validity of the interview instrument. Moreover, the first author's pre-understanding of the local context having been involved in TB/HIV management in the region, together with the rapport created with the participants during the time spent with them before the interviews, generated trust and encouraged free flow of the discussions. Notwithstanding, a well prepared interview guide was used as a means of avoiding preconceived ideas about the context and situation [12]. This measure was to explore new ideas and to ensure the discovery of new knowledge. During the analysis phase, many joint sessions were conducted with members of the research team to compare the codes, validate the interpretation of the data, and also to resolve any discrepancies in the observed findings. Finally, feedback of the findings was provided to the study hospitals and the relevant ethical bodies to ensure credibility of the results.

### Ethical considerations

Ethical approval for the study was obtained from the Regional Delegation of Public Health for the Northwest Region (N°401/NWP/PDPH/08). Administrative clearance was also obtained from the Internal Review Board of the Regional Hospital in Bamenda, and the Cameroon Baptist Convention Health Board Institutional Review Board (IRBC20090112ez: IRB2007-09), and St. Martin de Porres Catholic Hospital Njinikom. Verbal consent was obtained from each and every participant, and measures to safeguard their identities were explained to them.

## Results

A total of 32 participants were interviewed: 30 counsellors (24 females and 6 males), and 2 male traditional healers. The higher proportion of female counsellors compared to males in this study reflects that females make up the majority of the nursing profession in Cameroon. Their ages ranged from 27 to 65 years with a mean age of 36.5 years. Detailed characteristics of the participants are presented in Table 1.

**Table 1:** Characteristics of interview participants (N = 32)

Characteristic	Number
**Sex**	
Male	8
Female	24
	
**Professional background**	
Nurse/counsellor	16
Full time counsellor	4
Community relay agent/counsellor	5
Social worker/counsellor	5
Traditional healer	2
	
Counsellor training*	
< 1 week initial training + refresher courses	15
1-2 weeks initial training + refresher courses	10
1-2 months initial training + refresher courses	4
> 2 months initial training + refresher courses	1
	
Working experience*	
0-2 years	4
2 - 5 years	16
> 5 - 10 years	10

*Excluding two traditional healers

From the analysis, three themes that illustrate TB and HIV nurses/counsellors' experiences about counselling, delivering joint TB and HIV/AIDS services, and the constraints to effective collaboration between TB and HIV services emerged. The theme "Counselling is a call to serve humanity" is an illustration of the decision to become TB/HIV counsellors. Meanwhile, "Good training and devotion breed competence" is a reflection of the reasons for the improved uptake of counselling and testing services on account of the appropriate training received and the skills acquired from their work experience. Finally, the theme "Teamwork amidst adversity is the recipe for success" is an illustration of the reasons for the achievements in the collaboration and in addressing some of the constraints within both programmes. The themes are presented below with relevant quotations from the participants to elaborate on the findings.

### Counselling is a call to serve humanity

In general, many of the participants stated that the decision to become counsellors was personal. They mentioned that they had concern for the suffering and enjoyed talking to such patients. This was particularly so when it came to giving hope to the sick and dying.

*"It is because of my sympathy for humans. It is my joy when I assist people in need and I see some of them picking up health wise, I am very happy, that inspires me to go on" *(Female counsellor, 32 years old).

Meanwhile, others stated that it was because they had been working as TB nurses and saw the need to become counsellors considering the increasing number of TB patients that required HIV counselling. The motivating factors were therefore the desire for counselling skills and by that to reduce the number of patients referred to the HIV unit. In contrast, some participants declared that they were simply delegated by the hospital administration to undergo the counselling training because of insufficient qualified counsellors in the units. To this effect, some participants underscored the importance of delegating only staff that were motivated to work in both programmes considering the occupational risks and challenges of working in TB and HIV programmes.

*"Generally, they (nurses) don't like to work in the TB ward.....To make things worse they say they are risking already a lot for HIV and they cannot do an extra risk for TB without being compensated" *(Male nurse/counsellor, 33 years old).

Some participants in the faith-based hospitals further mentioned that some staff had confided to colleagues that they despised working in the units since it was against their wish. The participants declared that delegating such unmotivated staff could sometimes negatively impact their morals and productivity. To a few participants, training in counselling was an integral part of their formation as community relay agents working in TB and HIV programmes.

Regardless of the reasons of becoming counsellors, all the participants acknowledged that since embarking on the profession, they have found it gratifying and this has inspired them to continue helping other people to know more about TB and HIV, to make important decisions to protect themselves, to prevent infecting others and to receive appropriate/prompt treatment.

### Good training and devotion breed competence

The participants emphasised the importance of the initial detailed training in counselling which they received because it served as an eye-opener. The majority of them stated that they were initially trained for less than 2 weeks, with one exceptional case trained for 4 months (Table 1). The training was either organised by the government and/or their respective hospitals. Overall, they commended the training received despite the immense amount of material covered within the short period of training.

*"Overall it was good but I think there was too much information for the short time. We had to read and cover a lot of material during that period but it was good because we learned a lot" *(Female counsellor, 55 years old).

Although it was mentioned that refresher courses were organised either by the government or as part of in-service training, the participants declared that these have been infrequent. They therefore expressed the need for regular courses to adequately equip them against their professional challenges. Some stated that they had resorted to reading books, searching for relevant materials on the internet and learning informally from experienced colleagues in order to improve their knowledge and skills. All the participants ultimately acknowledged that the initial training complemented with the refresher courses and occasional supervision from their seniors have served as foundations for their professional life.

Regarding their counselling experience, the participants stated that at the onset they were apprehensive about convincing patients to accept testing for HIV. However, from the training and work experience gained over the years, there have been improvements in their interactions and communications with patients. They commented that this has been one of the reasons for the high HIV testing rate.

*"When we started... most of us were inexperienced. We never knew how to present most of the things to most of the patients but with the trainings; it has made most of the patients to be understanding" *(Male counsellor, 35 years old).

All the participants stressed that there is a minimum package of information they normally provide to patients during counselling. In pre-counselling, they mentioned that they ascertain the patients' knowledge of HIV and probe for misconceptions, provide information about the link between TB and HIV and the benefits of testing for HIV. In post-counselling, they explain the significance of the result and check for any misunderstanding, address the patients' psychological state and devise coping mechanisms, encourage patients to disclose their results to their loved ones, provide appropriate support services, discuss changes/reduction in risky behaviours, provide sexual and reproductive education, and discuss the possibility of ART. They also underscored the importance of informed consent.

*"HIV test is not really a must.... It is their [patients'] choice but we try as much as possible to continue letting them see the need for the test" *(Female counsellor, 30 years old).

Although they were sensitive to the patients' feelings, the participants declared that upholding patients' confidentiality and encouraging them to disclose their results, especially to their sexual partners, was challenging. Since respect for confidentiality is emphasised during their training and in their practice, it was a dilemma between respecting patients' right to confidentiality and disclosing their HIV status to their sexual partners at risk when they faced recalcitrant patients. Generally, they stated that confidentiality issues have been properly handled within their settings and this has enhanced the patients' trust in the healthcare system and improved uptake of services. However, some participants mentioned there have been investigations of isolated incidences of breach in confidentiality by some staff.

### Teamwork amidst adversity is the recipe for success

The participants highlighted the importance of working as a team to address some of the constraints in joint service delivery as one of the major reasons for the success achieved so far in the fight against TB and HIV in the region. This teamwork was directed to either colleagues within the healthcare setting or to other key stakeholders involved in TB and HIV care within the community.

#### Teamwork within the healthcare setting

The participants stated that collaboration between TB and HIV units has been beneficial both to the patients and the staff. The participants in faith-based centres mentioned that since both TB and HIV units were co-located, the staff actually conducted their activities in parallel. They affirmed this has greatly reduced the waiting time for patients in the hospital and improved patient monitoring.

*"I think it makes us to follow up our patients properly because among our patients we easily know which of them has TB or HIV. If we had separate units maybe we would not be able to follow them up well but since we operate in the same room we know them well" *(Female counsellor, 32 years old).

Some participants also remarked that since both TB and HIV patients regularly associate with each other in the same building, this has greatly reduced the stigma associated with both diseases thereby enhancing the uptake of TB and HIV services. Furthermore, they declared that it was the same medical officer that was in charge of both units, a strategy which has improved patient monitoring since the doctor is familiar with all the patients including those co-infected, and their treatment regimen. Participants in the public hospital mentioned that although TB and HIV units were located separately both physically and functionally due to lack of space, a therapeutic committee exists comprising of TB and HIV nurses/counsellors and doctors from both units. They met twice weekly to deliberate on the best ART regimen for newly diagnosed HIV-positive patients eligible for treatment, and to monitor the progress of old patients already on ART.

As regards benefits of collaboration to the staff, the participants in the faith-based centres stated that although they supposedly work in different units, they assist each other in their daily tasks. Some also commented that although they were not officially trained in handling both infections, they had broadened their knowledge and skills in handling co-infected patients by working with colleagues from the other unit. This they stated has made them more comfortable when rendering services to co-infected patients.

Despite the benefits of collaborating with colleagues, the respondents stated that they faced a number of constraints that adversely affected their work. All the participants articulated the problem of shortage of staff in the units compared to the heavy workload. Apart from performing counselling, they mentioned that they also carried out other duties in their respective units including dispensing drugs, record keeping, and assisting in the wards. Some participants from the faith-based centres said that they were sometimes delegated to assist in different units in the hospital. They all acknowledged that this increase in workload occasionally had a negative effect on their performance, especially when they are overwhelmed by constantly delivering HIV-positive results to patients. All the participants also raised the problem of shortage of counselling rooms which obliged them to use their offices to conduct other activities like consultation, drugs dispensing and documentation. Others stated that they shared office space with other colleagues and this limited the time they spent on counselling since they were compelled to show consideration for both their colleagues and other patients awaiting services. In one faith-based centre, the participants declared that both TB and HIV units shared the same office space and activities were carried out simultaneously by different counsellors. They acknowledged that such a situation could jeopardise patients' privacy/confidentiality. The problem of shortage of infrastructure also extended to the wards and outpatient waiting rooms in all the treatment centres where TB and HIV patients could occasionally be confined to the same rooms.

Another important constraint that the participants mentioned was frequent interruptions in drug supplies. They stated that supplies of anti-TB drugs and ARTs were fairly regular although there had been instances of temporary rupture of ART stocks for which they sought alternate supplies from neighbouring treatment centres. The most pertinent problem addressed was the complete rupture of CPT stocks and other drugs for treating HIV opportunistic infections which could last for several months. To solve this problem, some participants declared that they were obliged to ration CPT supplies only to newly diagnosed co-infected patients or to those who were severely immuno-compromised. However, they stated that they endeavoured to educate other patients on the importance of taking CPT regularly, and advised them to procure supplies at much subsidised rates in the hospital pharmacies. This was not without suspicion since they declared that some patients doubted their explanations for this measure.

*"... because we had told them it (CPT) was free so they expect us to give them because some of them think we have hidden it to sell to them later. We try to explain to them why it is not available, some understand but it is very difficult to convince them" *(Female counsellor, 32 years old).

#### Teamwork with the community

Although the participants acknowledged that uptake of TB/HIV services has greatly improved, they mentioned that they were still frustrated by some patients who refused to test for HIV. They cited the following as the main reasons for declining an HIV test: i) fear of HIV stigma, ii) associating TB diagnosis with HIV, iii) the lack of cure for HIV, iv) fear of lack of confidentiality of HIV results, v) fear of accusation by partners, and vi) the influence of some traditional healers who relentlessly dissuade the public that they can provide a permanent "cure" for HIV or that HIV is a spell cast on someone rather than an infection. Recognising the importance of collaborating with all stakeholders involved in TB/HIV prevention and control, the respondents asserted to have been working with community and religious leaders, youth peer educators and PLWHA to educate and sensitise the community about TB and HIV, and to fight stigma/discrimination. One participant declared to have been using the media for similar purposes. The respondents from one of the faith-based hospitals mentioned that they have been collaborating with traditional healers in TB/HIV prevention. What prompted this strategy was the realisation that many traditional healers professed to "cure" HIV/AIDS unlike mainstream medicine which only offers treatment. This message, the participants declared, has been the driving force behind the traditional healers' continued success in convincing patients to patronise them. Some respondent mentioned that there had been instances where patients who had dramatically improved while on ART, had abandoned their treatment in preference to traditional remedies only to return worse-off.

*"...one other big problem is when trying to convince our patients about traditional medicine, because many still use them...The man (traditional healer) told a patient that after one year the virus is going to clear from her system. She took the drug for more than a year. She was really down because when she came back her CD4 was 2" *(Female counsellor, 30 years old).

Two traditional healers interviewed admitted to have benefitted a lot from collaborating with one of the treatment centres for over 8 years. They acknowledged to have been trained by the treatment centre on recognising the signs and symptoms of HIV, and on HIV prevention/control. They also mentioned that they refer all suspected cases of HIV to the treatment centre using special referral forms, and they also document their activities in registers provided by the centre. Although they admitted to have received material support from the treatment centre to enhance their practice, they declared that it was important that they are regularly compensated for their efforts in ensuring a smooth collaboration, and they should also be provided with feedback about the patients they refer to the treatment centre. Reacting to allegations that traditional healers continue to undermine TB/HIV prevention and control, they emphasised that all registered members in their association who have been trained by the centre have acknowledged their limitations in connection with treating HIV. It was therefore the unregistered traditional healers who still ignorantly profess to cure HIV.

*"Those who do not attend our meetings doubt themselves and their activities. They claim they can "finish it" (cure HIV) meanwhile they cannot really explain how it (HIV) affects the person *(Traditional healer, 65 years old).

Although the nurses/counsellors interviewed in this study disclosed to have a good relationship with the TB/HIV patients, an important challenge which they mentioned was dealing with patients who do not adhere to treatment. Some of the reasons they cited for poor patient compliance included lack of money for follow-up visits, long distance or difficult terrain from the patients' residence to the hospital, and patients who resorted to economic activities like farming after having improved on treatment. Regarding patients with financial difficulties, some participants declared that they occasionally provide transport money either from private, hospital or donor funds when they are available. In addition, they have been educating patients on the importance of good treatment adherence and respecting their clinic appointments. The respondents also indicated to have extended this education to the patients' relatives and the community in general, informing them about the dangers of not encouraging patients to respect their hospital appointments.

Addressing HIV-positive patients' psychological and material needs was another big challenge mentioned by the participants since these could affect patients' treatment follow-up and adherence. They declared to have a close working relationship with social workers and chaplains since they regularly refer patients with psychosocial and spiritual needs to these services. Besides, they stated that they refer newly diagnosed HIV patients to HIV support groups that were created by the treatment centres. This strategy is to encourage interactions between previously diagnosed HIV-positive patients who have been educated to provide peer support to new members in the groups, and to assist the new members to commence income-generating activities that will enable them to become self-sufficient. Observations of activities between members of two HIV support groups created by one of the faith-based hospitals revealed that they are regularly educated on how to live positively with their HIV-positive status, to support each other, to make lifestyle adjustments and prevent HIV transmission, on good hygiene and nutrition, and treatment compliance. It was also observed that members have benefitted from funds to engage in income generating activities corroborating the statements of the counsellors in the treatment centres. Some members in the support groups acknowledged to have been trained on counselling, and were serving as "expert patients", providing HIV counselling to patients suspected of having HIV as a strategy to address staff shortage in the treatment centre. Other activities which members of the support groups performed included conducting home visits to sick members in the community, providing public sensitisation campaigns in schools and in the communities to encourage early health seeking and to fight stigma in the society. In one support group, it was observed that members were registered with a community health insurance scheme which covered the cost of their laboratory investigations associated with HIV treatment and follow-up.

## Discussion

This study highlights the perspectives/experiences of TB and HIV nurses/counsellors who serve as frontline actors in implementing TB and HIV collaborative activities in the Northwest region of Cameroon. The discussion is structured in three sections: i) counselling training and practice, ii) teamwork with important stakeholders directly or indirectly involved with TB and HIV control, and iii) health system constraints. Each section addresses specific concerns which warrant due consideration when scaling up TB and HIV collaborative activities in order to ensure success and sustainability.

### Counselling training and practice

Our study revealed that the majority of the participants' motivation to become counsellors was based on humanitarian grounds. Nonetheless, some were selected not because of their interest in the profession but simply as a measure to address the shortage of counsellors in the centres, a finding consistent with other studies [13,14]. However, such a measure could be counterproductive in the long run since such staff lack the dedication to perform their duties, a situation which could jeopardise good service delivery. Moreover, the initial quality and duration of training the participants received were variable, and coupled with the fact that ongoing in-service training and supportive supervision were infrequent; this caused apprehension among some participants when they attended to patients co-infected with TB and HIV. It was also a dilemma to the respondents between upholding confidentiality of patients' results and encouraging voluntary disclosure of HIV status to patients' sexual partners when faced with uncompromising patients. All these challenges could adversely affect the counsellors' morals leading to burnout as has been documented in other research [13]. These concerns therefore underscore the importance of addressing human resources development by identifying and selecting interested staff for training, providing regular in-service refresher courses/seminars, and supportive supervision for the staff in the treatment centres. In addition, counsellor support groups should be encouraged which will provide a forum for members to meet and discuss their daily challenges and seek strategies to improve joint service delivery.

### Teamwork with key TB and HIV/AIDS stakeholders

Our study revealed that all the participants appreciated the integration of TB and HIV services because it was beneficial both to the staff and, most importantly, to the patients. The achievements in collaboration were attributed to the multidisciplinary approach to service delivery since staff from both TB and HIV units collaborated with each other, with colleagues in other treatment centres, with the patients and HIV support group members, and with key stakeholders in the community. Staff from both units assisted each other although they had specific duties to perform in their respective units. Teamwork was feasible because of the cross-training in TB and HIV which staff from both units received either formally or informally. This enabled some of them to become conversant with the delivery of both TB and HIV services, consistent with the finding from another study in Tanzania [15]. Additionally, the existence of a therapeutic committee in one of the treatment centres ensured that co-infected patients were properly treated and monitored by a team from both units. All these measures were demonstrations of collaboration which improved joint TB and HIV service delivery.

The teamwork demonstrated in this study also existed outside the spheres of the healthcare setting, between the healthcare providers, the patients and the community to address issues of poor compliance to treatment through education and sensitisation. Since poverty was cited as one of the reasons for poor or non-compliance to treatment, study participants occasionally provided financial assistance to paupers. However, a more sustainable intervention to tackle poverty and address the psychosocial needs of these patients was to refer them to HIV support groups affiliated to the treatment centres where they received peer support and counselling, and most especially empowering them economically with subsidies to engage in income-generating activities. Consistent with some of the findings in our study and also documented in other settings, HIV support groups have been recognised to increase patients' access to HIV services, to foster patient unity, to fight stigma and discrimination, and to provide peer education. Moreover, the groups provide patients with funds to commence income-generating activities to ensure self-subsistence and ultimately improve patients' treatment compliance and outcomes [16-20]. The experiences from members of the support groups and healthcare staff overseeing these groups could therefore be valuable to managers in other treatment centres and the health system in general when considering creating or scaling up HIV support groups. Another interesting strategy that addressed HIV-positive patients' health expenditures was the creation of a community insurance which is a form of prepayment and risk-pooling scheme which registered support group members. This scheme catered for HIV support group members' HIV therapeutic investigations. Such a scheme could be rolled out nationwide and in other African settings with high HIV prevalence considering the high cost of out-of-pocket health financing in these settings [21].

There has been documented reluctance of mainstream medicine to collaborate with traditional healers in Africa [22]. However, an interesting finding in this study was the collaboration that existed regarding HIV prevention and control between one of the faith-based centres and some traditional healers operating within the treatment centre's vicinity. Another study in Cameroon also revealed traditional healers' willingness to collaborate with the mainstream healthcare system [23]. Notwithstanding, our study also indicated that traditional healers continue to undermine TB and HIV/AIDS prevention and control activities in the region, a position that was corroborated by the traditional healers interviewed in this study. Traditional medical practice has been legalised by the Cameroon government considering their huge influence in the population since they usually serve as entry points into the healthcare system. It has been estimated that 80% of people in Africa rely on traditional medicine for their primary health needs [24]. It would therefore be indispensible to harness traditional healers' potentials by streamlining them into TB/HIV prevention and control activities nationwide since our study and other studies in some African countries [25] have demonstrated that this is feasible. Creating a forum which recognises all traditional medical practitioners involved in TB/HIV activities in the region, and providing them with the basic training and follow-up supervision on TB/HIV prevention, control and referral of suspect cases would be invaluable. However, the concerns which the traditional healers raised in this study should be duly considered because treating TB and HIV/AIDS patients is part of their livelihood, and for which they could regard as being threatened if these groups of patients are taken away from them into mainstream medicine.

### Health system constraints

Despite the achievements mentioned above towards achieving a successful collaboration between TB and HIV service delivery, the respondents highlighted important constraints which could jeopardise the existing efforts. A pertinent subject discussed in our study was the shortage of staff in both units compared to the heavy workload. Staff shortages as well as inadequately trained staff providing integrated TB/HIV services have also been reported in South Africa and Uganda [14,26,27]. In our study, understaffing necessitated that participants performed multiple duties including counselling, a situation which could adversely affect their efficiency. Moreover, in order to increase patient involvement in care, and improve HIV testing rates, staff shortage concerns also prompted some centres to use "expert patients" who were members of HIV support groups whom had been trained to serve as lay volunteer counsellors. There is evidence that task-shifting is an effective strategy to address the immediate shortage of human resources in HIV treatment and care in Africa [28]. The Cameroon government had initiated the community relay agent programme, recruiting and training staff in integrated TB, HIV and Malaria care. Subsequently, these staff were to serve as bridges between the patients in the community and the healthcare system. However, there have been challenges regarding appropriate remuneration of these agents and sustainability of this programme. Strong political commitment is therefore required to gradually scale up the use of such lay counsellors to address the immediate shortages of human resources within treatment centres. Nevertheless, the contextual factors which might affect such a strategy require due consideration. Additionally, the roles of such workers within the healthcare system have to be clearly defined.

It was observed in this study that TB and HIV units were co-located in the faith-based centres with the same clinician overseeing patient care, similar to the Khayelitsha model in South Africa [26]. Such a strategy enhances proximity of the units and reduces the possibility of duplication of services which ultimately improves service quality and delivery. However, due to insufficient space in the public hospital, TB and HIV units still operated in separate buildings. This could hamper inter-unit referral of co-infected patients and loss to follow-up within the system if proper tracking and referral mechanisms are not put in place. Lack of infrastructure as a general problem within treatment centres has been documented [14]. Privacy and confidentiality concerns, and delay in service provision as a result of inadequate work space were also raised in this study. Improvement in health system infrastructure is required to address these constraints, while appropriate infection control measures should be put in place to prevent the spread of nosocomial TB infection to HIV-positive patients who are high risk [29] as a result of their exposure to TB patients in cramped outpatient waiting rooms and in the wards.

It is a commendable finding in this study that anti-TB medications and ART supplies have been fairly regular in all the treatment centres with only minor and short-term incidences of interrupted supplies of ARTs. Such incidences were duly addressed by staff that requested for relief supplies from nearby treatment centres with "surplus" stocks. Such a strategy is a demonstration of the healthcare staff's ingenuity in addressing challenges and which could be scaled-up by creating a network of all TB and HIV treatment centres operating in the region and their corresponding staff as has been reported in a South African study [13]. This will ensure timely request of relief supplies and the possibility of referring patients to treatment centres with "adequate" stocks if located close to the patients' area of residence. However, a distressing finding in this study was the fact that CPT stocks had been interrupted for several months and consistent with study reports from Uganda [14]. This resulted in the rationing of CPT only to newly diagnosed and/or severely immunocompromised HIV patients, meanwhile other HIV-positive patients were expected to personally procure their supplies. In Cameroon, household out-of-pocket health expenditure has been documented to be over 80% [30]. Asking patients who are already financially incapacitated would be catastrophic and could lead to poor treatment compliance or non-compliance. It is therefore imperative that a consistent and sustainable drug supply mechanism is put in place considering the benefits of this simple but effective preventive strategy [31].

#### Methodological considerations

The study hospitals were purposefully selected because they are approved TB and HIV treatment centres with up-to-date laboratory monitoring facilities, and they also serve as referral centres in the region. We therefore believe that they share similar characteristics and challenges as the other smaller treatment units in the region, and therefore our results could be applicable to the entire region and even on the national level. However, inherent in the study design and sampling, the participants' perceptions do not necessarily represent those of the entire nurses/counsellors and traditional healers in the region or the entire country. Additionally, the first author has been involved in TB/HIV care in the region and was familiar to the participants. This might have influenced their responses either by them providing less information assuming that the interviewer was already familiar with the settings or they could have provided favourable responses to highlight their successes despite all the measures taken to reduce this bias. Finally, although our findings might be context-specific, the lessons learned from this study can inform implementation or strengthening of TB and HIV collaborative activities in similar settings with high co-infection rates which is the case with most countries in sub-Saharan Africa.

## Conclusions

In the past, TB and HIV programmes have provided their services as separate entities. However, the synergism between TB and HIV/AIDS has prompted collaboration between both programmes. Our study highlights the importance of adequate planning and investment/strengthening of the health system while scaling up collaborative activities into already existing programmes. These measures are pertinent since shortages in human resources and infrastructure, and interrupted supply of drugs remain important constraints to successful teamwork. A multidisciplinary approach to TB/HIV prevention and control involving key stakeholders is essential. Particular consideration should be given to the enormous potential that could be harnessed by collaborating with traditional healers while clearly defining their roles in the partnership. Finally, sustainable interventions to address poverty in this particularly disadvantaged group of patients would be invaluable while providing them with appropriate psychosocial support to encourage health seeking and treatment adherence.

## Competing interests

The authors declare that they have no competing interests.

## Authors' contributions

All authors contributed to the paper. BNN and AKH conceptualised and designed the study; BNN performed data collection and analysis which was interpreted by all authors. BNN drafted the manuscript with substantial revisions from all authors. All authors read and approved the final version of the manuscript.

## Pre-publication history

The pre-publication history for this paper can be accessed here:

http://www.biomedcentral.com/1472-6963/11/328/prepub
